# Cyclic ADP Ribose-Dependent Ca^2+^ Release by Group I Metabotropic Glutamate Receptors in Acutely Dissociated Rat Hippocampal Neurons

**DOI:** 10.1371/journal.pone.0026625

**Published:** 2011-10-20

**Authors:** Jong-Woo Sohn, Weon-Jin Yu, Doyun Lee, Hee-Sup Shin, Suk-Ho Lee, Won-Kyung Ho

**Affiliations:** 1 Department of Physiology and Biomembrane Plasticity Research Center, Seoul National University College of Medicine, Seoul, Korea; 2 Center for Neural Science, Korea Institute of Science and Technology, Seoul, Korea; Indiana University School of Medicine, United States of America

## Abstract

Group I metabotropic glutamate receptors (group I mGluRs; mGluR1 and mGluR5) exert diverse effects on neuronal and synaptic functions, many of which are regulated by intracellular Ca^2+^. In this study, we characterized the cellular mechanisms underlying Ca^2+^ mobilization induced by (*RS*)-3,5-dihydroxyphenylglycine (DHPG; a specific group I mGluR agonist) in the somata of acutely dissociated rat hippocampal neurons using microfluorometry. We found that DHPG activates mGluR5 to mobilize intracellular Ca^2+^ from ryanodine-sensitive stores via cyclic adenosine diphosphate ribose (cADPR), while the PLC/IP_3_ signaling pathway was not involved in Ca^2+^ mobilization. The application of glutamate, which depolarized the membrane potential by 28.5±4.9 mV (n = 4), led to transient Ca^2+^ mobilization by mGluR5 and Ca^2+^ influx through L-type Ca^2+^ channels. We found no evidence that mGluR5-mediated Ca^2+^ release and Ca^2+^ influx through L-type Ca^2+^ channels interact to generate supralinear Ca^2+^ transients. Our study provides novel insights into the mechanisms of intracellular Ca^2+^ mobilization by mGluR5 in the somata of hippocampal neurons.

## Introduction

The group I metabotropic glutamate receptors (mGluRs), which include mGluR1 and mGluR5, play important roles in regulating intrinsic excitability and synaptic plasticity [1], [2], [3]. Importantly, intracellular Ca^2+^ contributes to various aspects of mGluR-mediated effects. Enhancement of neuronal excitability [4], [5], [6] and long-term depression mediated by mGluR (mGluR-LTD) [7] were shown to be blocked by intracellular dialysis of BAPTA, and the involvement of Ca^2+^-dependent proteins such as PICK1 and NCS-1 in mGluR-LTD has recently been demonstrated [8], [9], [10]. In addition, mGluR triggers retrograde endocannabinoid signaling, an effect that is greatly enhanced by increases in Ca^2+^ [11], [12]. However, the signaling pathways and the source of Ca^2+^ that contributes to these diverse effects have not yet been clearly elucidated.

It is well known that group I mGluRs mobilize Ca^2+^ from intracellular stores in hippocampal neurons [13], [14]. As group I mGluRs are coupled to Gq proteins [15], [16], Ca^2+^ mobilization may involve the phospholipase C (PLC)/inositol-3-triphosphate (IP_3_) signaling pathways [1]. Indeed, the synergistic or supralinear Ca^2+^ release by group I mGluR stimulation paired with backpropagating action potential (AP) was shown to be from IP_3_ receptor (IP_3_R)-sensitive intracellular stores in apical dendrites of CA1 hippocampus [17], [18]. However, studies in midbrain dopaminergic neurons demonstrated that intracellular Ca^2+^ mobilization by group I mGluRs required cyclic ADPR ribose (cADPR)/ryanodine receptors (RyRs) as well as IP_3_/IP_3_Rs [19]. The role of cADPR in mGluR-mediated Ca^2+^ signaling is supported by the study showing that the glutamate-induced stimulation of ADP-ribosyl cyclase occurs preferentially in NG108-15 neuroblastoma/glioma hybrid cells over-expressing mGluR1, 3, 5, and 6 [20]. It is not yet clear if this finding could also be extended to hippocampal neurons, but considering the frequent involvement of PLC-independent signaling pathways in several effects of group I mGluRs [21], [22], [23], [24], the possibility that Ca^2+^ mobilization by group I mGluR may be mediated by signal pathways other than PLC/IP_3_Rs should be tested in hippocampal neurons.

Ca^2+^ signaling in neurons is highly compartmentalized, with Ca^2+^ having distinctive roles in each section [25], [26], [27]. Mechanisms involved in axonal and dendritic Ca^2+^ signaling have been extensively studied due to their importance in the regulation of neurotransmitter release and synaptic plasticity [28], [29], [30]. Somatic Ca^2+^ signals also play important roles in regulating cellular excitability, synaptic plasticity and gene expression [7], [31], [32], but the mechanisms involved in somatic Ca^2+^ signals are not well studied. As different neuronal compartment may have distinct Ca^2+^ signaling machinery, results obtained from dendrites or axons may not extend to the somatic Ca^2+^ signals. Therefore, separate studies of somatic Ca^2+^ signals are warranted.

In the current study, we directly investigated the signaling pathways underlying somatic Ca^2+^ mobilization by group I mGluRs. Using microfluorometric Ca^2+^ measurements in the somata of acutely dissociated rat hippocampal neurons loaded with Fura 2-AM, we discovered that stimulation of group I mGluRs induces the cADPR-dependent Ca^2+^ mobilization from ryanodine-sensitive stores. Our results represent a novel mechanism for Ca^2+^ mobilization by group I mGluRs in hippocampal neurons.

## Results

### mGluR5 is responsible for DHPG-induced Ca^2+^ release from intracellular stores

To investigate the mechanisms underlying Ca^2+^ increase by group I mGluR stimulation, acutely dissociated hippocampal CA1 neurons were loaded with 2 µM Fura 2-AM for microfluorometry experiments. The application of 50 µM (*RS*)-3,5-dihydroxyphenylglycine (DHPG), a specific group I mGluR agonist, to these cells rapidly increased intracellular Ca^2+^ concentrations in the somata. The amplitude of DHPG-induced Ca^2+^ increase (Ca_DHPG_) was highly variable among cells, ranging from ∼20 nM to ∼500 nM (mean = 97.5±7.8 nM, n = 168), but Ca_DHPG_ values obtained from the same cell upon repetitive application of DHPG at 2 min intervals yielded consistent data (Ca_DHPG,2_/Ca_DHPG,1_ = 98.6±5.1%, n = 6). To investigate the mechanism of DHPG-induced Ca^2+^ increase, we regarded Ca_DHPG,1_ as the control and applied various experimental conditions prior to the second application of DHPG. The relative amplitude of Ca_DHPG,2_ compared with Ca_DHPG,1_ (Ca_DHPG,2_/Ca_DHPG,1_) was obtained to study the contribution of each variable to the DHPG-induced Ca^2+^ increase.

**Figure 1:**
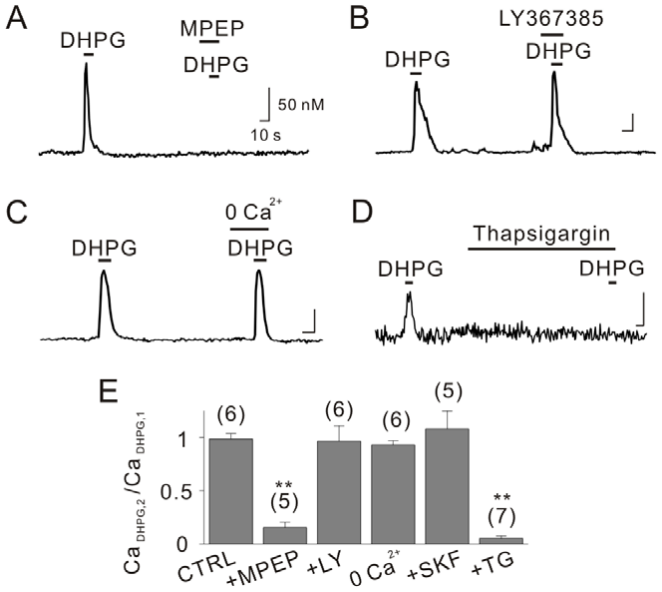
mGluR5 activation by DHPG leads to intracellular Ca^2+^ mobilization. Acutely dissociated rat hippocampal CA1 neurons were loaded with 2 µM Fura 2-AM for Ca^2+^ measurements. Cells were pretreated with 25 µM MPEP (A), 100 µM LY367385 (B), 0 Ca^2+^ external solutions (C) or 2 µM thapsigargin (D) prior to the second application of DHPG (50 µM). (E) Bar graphs represent the relative peak amplitude of second Ca^2+^ transients to those of first (Ca_DHPG,2_/Ca_DHPG,1_). Scale bars indicate 10 sec (horizontal) and 50 nM (vertical). CTRL = control, LY = LY367385, SKF = SKF96365, TG = thapsigargin. ** indicates *p*<0.01.

The amplitude of the second Ca^2+^ transient was significantly suppressed by the selective mGluR5 antagonist, MPEP (25 µM), but not by the mGluR1 antagonist LY367385 (100 µM), indicating that mGluR5 is responsible for the DHPG-induced Ca^2+^ increases in hippocampal CA1 neurons (Figure 1A & 1B). DHPG-induced Ca^2+^ transients were not affected by the removal of external Ca^2+^ or the inhibition of receptor-operated Ca^2+^ entry by SKF96365 (10 µM), but they were markedly suppressed when cells were pretreated with the sarcoplasmic/endoplasmic reticulum Ca^2+^-ATPase (SERCA) inhibitor thapsigargin (2 µM) for 5 min, indicating that DHPG mobilizes Ca^2+^ from its intracellular stores (Figure 1C–1E).

### mGluR5-induced Ca^2+^ release from intracellular stores is independent of PLC-IP_3_

**Figure 2:**
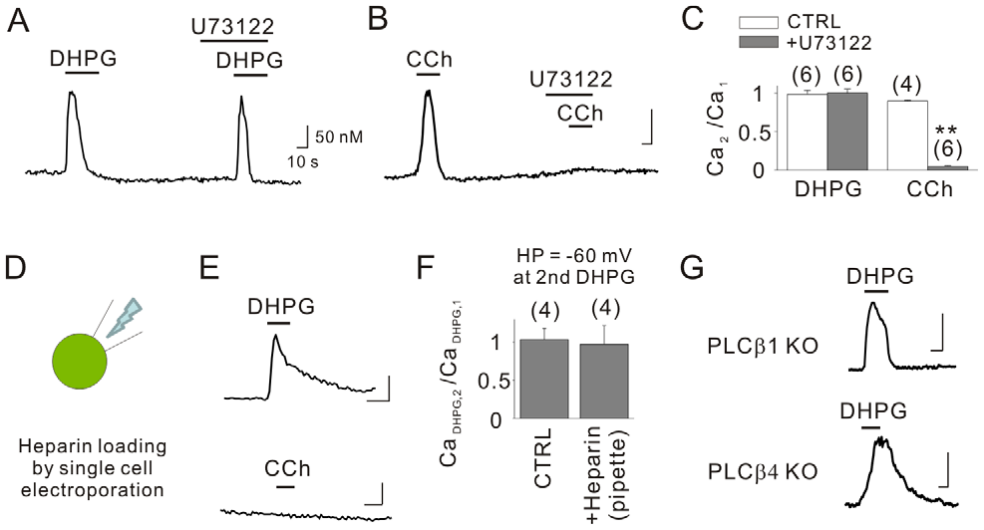
PLC/IP_3_R signaling pathways are not involved in DHPG-induced Ca^2+^ mobilization. U73122 (1 µM), a PLC inhibitor, did not inhibit induction of Ca^2+^ transients by DHPG (A), but blocked those induced by the muscarinic receptor agonist CCh (10 µM, B). (C) The Ca_2_/Ca_1_ ratio was 90.0±0.9% (n = 4) for CCh. It was 100.4±5.3% (n = 6) for DHPG and 4.8±1.0% (n = 6) for CCh when pretreated with U73122. (D) Cells were loaded with heparin (20 mg/ml) by single cell electroporation. Alexa Fluor-488 was added to the pipette solutions to confirm successful loadings. (E) CCh-induced Ca^2+^ transients were almost completely inhibited in cells loaded with heparin, a competitive antagonist of the IP_3_Rs, whereas DHPG still caused Ca^2+^ transients in cells loaded with heparin. (F) The Ca_DHPG,2_/Ca_DHPG,1_ ratio was 97.1±24.6% (n = 4) and 103.2±14.7% (n = 4) when cells were patched with pipette solutions with or without heparin and were voltage-clamped at −60 mV at the second DHPG application. (G) DHPG still induced Ca^2+^ transients in cells isolated from PLCβ1 (upper) or PLCβ4 (lower) knockout mice. Scale bars indicate 10 sec (horizontal) and 50 nM (vertical). ** indicates *p*<0.01.

We next tested whether the PLC/IP_3_ signaling pathways link mGluR5 and Ca^2+^ mobilization. Interestingly, DHPG-induced Ca^2+^ release was not affected by the PLC inhibitor U73122 (1 µM; Figure 2A & 2C). Conversely, muscarinic receptor-mediated Ca^2+^ transients induced by the muscarinic receptor agonist carbachol (CCh; 10 µM) were completely inhibited by U73122 (Figure 2B & 2C), confirming that U73122 effectively inhibited the PLC pathway under our experimental conditions. Subsequently, we loaded intact cells with heparin (20 mg/ml in the electroporation pipette), a competitive antagonist of the IP_3_Rs [33], using a single-cell electroporator. Loading of heparin was confirmed by co-administration of the fluorescent compound Alexa Fluor-488 (Figure 2D). This manipulation completely inhibited the induction of Ca^2+^ transients by CCh, but not those by DHPG (Figure 2E). For quantitative analyses, we measured the first DHPG-induced Ca^2+^ transient in a Fura 2-AM-loaded neuron, patched the same neuron with a Fura 2 (10 µM)-containing pipette with or without heparin (1 mg/ml), and re-applied DHPG to elicit a second Ca^2+^ transient. We confirmed that the amplitudes of the second Ca^2+^ transients, which were measured at a holding potential of −60 mV, did not differ from those of the first Ca^2+^ transients (103.2±14.7%, n = 4) (Figure 2F, left bar). The inclusion of heparin did not affect DHPG-induced Ca^2+^ transients (97.1±24.6%, n = 4) (Figure 2F, right bar).

PLCβ1 and PLCβ4 are known to mediate group I mGluR signaling in the brain [34], [35]; therefore, we tested whether DHPG induces Ca^2+^ transients in hippocampal CA1 neurons isolated from mice lacking the PLCβ1 or PLCβ4 subunits. As illustrated in Figure 2G, DHPG was still able to induce Ca^2+^ transients in cells from PLCβ1 or PLCβ4 knockout mice. These results suggest that mGluR5 induces Ca^2+^ release independently of PLC/IP_3_ signaling pathways.

### mGluR5 activates cADPR pathways to induce RyR-dependent Ca^2+^ release from intracellular stores

Alternatively, cADPR, which is metabolized from nicotinamide adenine dinucleotide (NAD^+^) by ADP-ribosyl cyclases [36], [37], [38], may be involved in mobilizing Ca^2+^ from intracellular stores. The involvement of ADP-ribosyl cyclase and/or cADPR in agonist-induced intracellular Ca^2+^ mobilization has been described in a variety of cell types [39], [40], [41], [42], [43], [44], [45], [46]. Notably, overexpression of mGluR1 or mGluR5 in NG108-15 cells induced the activation of ADP-ribosyl cyclase [20]. In addition, group I mGluR-induced Ca^2+^ release from midbrain dopaminergic neurons was shown to be mediated by both IP_3_ and cADPR [19]. Therefore, we tested the involvement of cADPR signal pathways in DHPG-induced Ca^2+^ mobilization.

**Figure 3:**
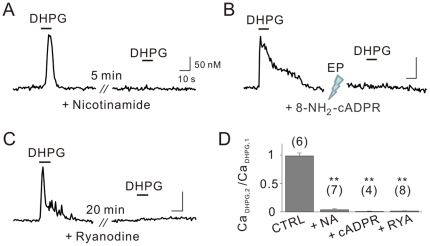
DHPG-induced intracellular Ca^2+^ mobilization occurs via the cADPR/RyR signaling pathways. DHPG-induced Ca^2+^ transients were completely inhibited when cells were pretreated with 5 mM nicotinamide (A), 100 µM 8-NH_2_-cADPR (B), or 20 µM ryanodine (C) prior to the second application of DHPG. (D) Bar graphs represent the relative peak amplitude of second Ca^2+^ transients to those of first (Ca_DHPG,2_/Ca_DHPG,1_). Scale bars indicate 10 sec (horizontal) and 50 nM (vertical). EP = electroporation, NA = nicotinamide, cADPR = 8-NH_2_-cADPR, RYA = ryanodine. ** indicates *p*<0.01.

When cells were pretreated with nicotinamide (5 mM) for 5 min to inhibit ADP-ribosyl cyclase [47], DHPG was no longer able to induce the production of Ca^2+^ transients (Figure 3A). Similarly, in single-cell electroporation experiments, DHPG failed to mobilize Ca^2+^ in neurons loaded with 8-NH_2_-cADPR (100 µM), a competitive antagonist of cADPR [19] (Figure 3B). Considerable evidence suggests that cADPR is the endogenous modulator of RyRs [45], [46], [48], [49], [50], [51]. Pretreating cells with ryanodine (20 µM) for 20 min [52] completely blocked the induction of Ca^2+^ transients by DHPG (Figure 3C). Importantly, DHPG-induced Ca^2+^ transients were completely abolished in every cell tested for these pharmacological inhibitors. These results indicate that mGluR5 activates cADPR signaling pathways to mobilize Ca^2+^ from ryanodine-sensitive stores in hippocampal neurons (Figure 3D).

### Glutamate-induced Ca^2+^ influx is mediated by L-type Ca^2+^ channels activated by AMPA receptor-mediated depolarization

**Figure 4:**
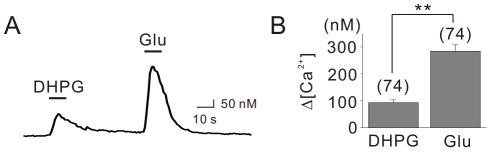
Glutamate-induced Ca^2+^ transients are significantly larger than DHPG-induced Ca^2+^ transients. (A) Cells were treated with both DHPG (50 µM) and glutamate (30 µM). (B) Bar graphs summarizing the average amplitudes of DHPG- and glutamate-induced Ca^2+^ transients from 74 cells. Scale bars indicate 10 sec (horizontal) and 50 nM (vertical). Glu = glutamate. ** indicates *p*<0.01.

Our results demonstrated a predominant role for the cADPR/RyR signaling pathway in mGluR5-induced Ca^2+^ release in the somata of hippocampal neurons. In the brain, glutamate is the natural neurotransmitter that stimulates mGluRs; glutamate can activate both mGluRs and ionotropic glutamate receptors (iGluRs), which include AMPA and NMDA receptors. NMDA receptors and the Ca^2+^-permeable AMPA receptors are possible sources of Ca^2+^ entry into the cell. In addition, glutamate-induced membrane depolarization should activate voltage-gated Ca^2+^ channels (VGCCs) to cause Ca^2+^ influx. Indeed, we confirmed that glutamate depolarizes the membrane potential by 29.0±4.6 mV (n = 4). As shown in Figure 4A, the applications of DHPG and glutamate (30 µM) on the same cell demonstrated that the amplitude of glutamate-induced Ca^2+^ transients (Ca_Glu_) was significantly greater than Ca_DHPG_. The average Ca_DHPG_ was 92.8±10.3 nM from 74 cells, whereas Ca_Glu_ was 284.3±23.6 nM from the same population (Figure 4B, p<0.01). The Ca_DHPG_/Ca_Glu_ ratio was calculated to be 39.0±3.6% (n = 74).

**Figure 5:**
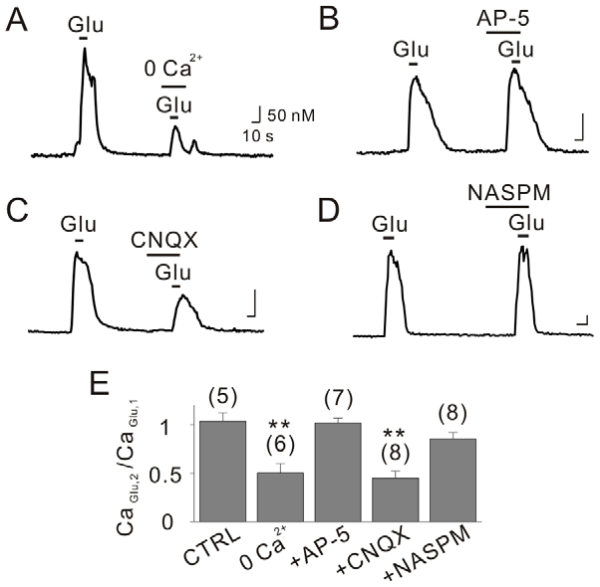
AMPA receptors, but not NMDA receptors, are responsible for glutamate-induced Ca^2+^ influx. (A) The amplitudes of glutamate-induced Ca^2+^ transients (Ca_Glu_) were significantly attenuated in Ca^2+^-free solutions. Ca_Glu_ was not affected by AP-5 (B), but was significantly decreased by the pretreatment with CNQX (C). (D) Ca_Glu_ was not inhibited by NASPM. (E) Bar graphs represent the ratio between first and second Ca_Glu_. Scale bars indicate 10 sec (horizontal) and 50 nM (vertical). ** indicates *p*<0.01.

Subsequently, experiments were performed to determine the source of Ca^2+^ entry when cells were stimulated with glutamate. Repetitive application of glutamate at 2 min intervals yielded reproducible Ca_Glu_ values (Ca_Glu,2_/Ca_Glu,1_ = 103.6±8.7%, n = 5). When the bath solution was replaced with a Ca^2+^-free solution prior to the second application of glutamate, the Ca_Glu,2_ was significantly decreased so that the Ca_Glu,2_/Ca_Glu,1_ ratio was 50.4±9.6% (n = 6, Figure 5A). Unexpectedly, the addition of the NMDA receptor blocker AP-5 before the second application of glutamate had no effect, and the Ca_Glu,2_/Ca_Glu,1_ ratio was 101.7±5.0% (n = 7, Figure 5B). In contrast, the addition of the AMPA receptor blocker CNQX prior to the second application of glutamate was as effective as the removal of Ca^2+^, producing a Ca_Glu,2_/Ca_Glu,1_ ratio of 45.1±7.1% (n = 8, Figure 5C), suggesting that AMPA receptors may be involved in glutamate-induced Ca^2+^ influx. However, the addition of 1-naphthyl acetyl spermine (NASPM, 10 µM), a specific blocker of the Ca^2+^-permeable AMPA receptor, had no significant effect on glutamate-induced Ca^2+^ transients (Figure 5D). These results suggest that neither NMDA nor AMPA receptors are involved in the observed Ca^2+^ influx pathway, but that AMPA receptor activation may trigger Ca^2+^ influx through VGCCs by depolarizing the membrane potential. In support of this, we found that glutamate-induced membrane depolarization was 5.0±1.2 mV (n = 3) in the presence of CNQX, which is significantly less than that observed in the control (29.0±4.6 mV, n = 4, p<0.05).

**Figure 6:**
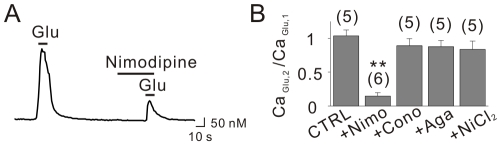
L-type Ca^2+^ channels are responsible for glutamate-induced Ca^2+^ influx. (A) Ca_Glu_ was greatly inhibited in the presence of nimodipine. (B) Bar graphs represent the ratio between first and second Ca_Glu_. Scale bars indicate 10 sec (horizontal) and 50 nM (vertical). Nimo = nimodipine, Cono = ω-conotoxin GVIA, Aga = ω-agatoxin IVA. ** indicates *p*<0.01.

To identify the subtype of VGCC responsible for the calcium influx, we tested the effects of specific pharmacological inhibitors. We found that nimodipine (10 µM), an L-type Ca^2+^ channel blocker, significantly reduced glutamate-induced Ca^2+^ transients (Figure 6A). In contrast, the amplitude of Ca_Glu_ was not significantly affected by ω-conotoxin GVIA (1 µM), ω-agatoxin IVA (200 nM), and NiCl_2_ (100 µM), indicating that N-type, P/Q type, T-type and R-type Ca^2+^ channels are not involved (Figure 6B). These data indicate that in glutamate application experiments L-type Ca^2+^ channels mediate Ca^2+^ entry triggered by AMPA receptor-mediated depolarization.

### cADPR/RyR-dependent Ca^2+^ release does not interact with the Ca^2+^ influx through L-type Ca^2+^ channels

To understand the complexity of mGluR-mediated Ca^2+^ signaling, it is necessary to examine the interaction between mGluR-induced Ca^2+^ release and glutamate-induced Ca^2+^ influx. It has been shown that Ca^2+^ entry through VGCCs interacts synergistically with IP_3_ to enhance mGluR-mediated Ca^2+^ release in apical dendrites of hippocampal CA1 neurons [17], [18]. Supralinear Ca^2+^ release by DHPG along with either membrane potential depolarization or NMDA receptor activation was also demonstrated in primary cultured hippocampal neurons [13]. Conversely, Topolnik et al. (2009) demonstrated that dendritic L-type Ca^2+^ channels are enhanced by mGluR5-induced Ca^2+^ mobilization from ryanodine-sensitive stores in the GABAergic interneurons of the hippocampus [53]. Notably, cADPR was shown to enhance L-type Ca^2+^ channels induced by both orthograde and retrograde pathways in NG108-15 cells [54]. Therefore, we analyzed Ca^2+^ transients by DHPG and glutamate, under the assumption that Ca_Glu_ represents the sum of Ca^2+^ influx (Ca_Influx_), Ca^2+^ mobilization by mGluR5 (Ca_DHPG_) and the supralinear Ca^2+^ transients (Ca_SUP_) by synergistic effects of mGluR5 and Ca^2+^ influx (Ca_Glu_ = Ca_Influx_+Ca_DHPG_+Ca_SUP_).

**Figure 7:**
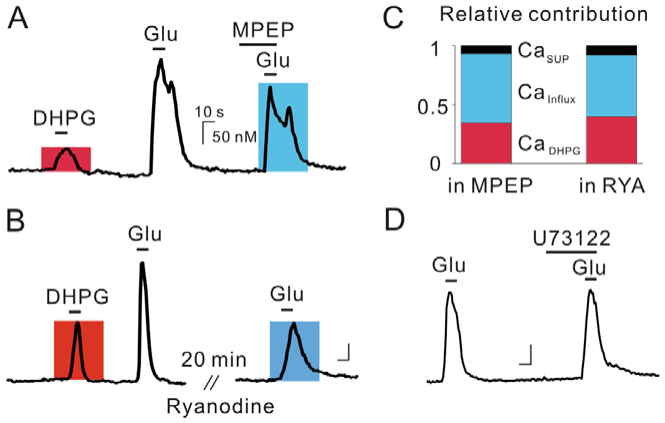
Glutamate-induced Ca^2+^ influx does not interact with DHPG-induced Ca^2+^ mobilization. (A) Ca^2+^ influx by glutamate was determined by comparing the amplitudes of two glutamate-induced Ca^2+^ transients in the presence or absence MPEP, which blocks DHPG-induced Ca^2+^ mobilization (blue box), and was compared to Ca^2+^ mobilization (red box). (B) Ca^2+^ influx by glutamate was determined by comparing the amplitudes of two glutamate-induced Ca^2+^ transients in the presence or absence of ryanodine, which blocks DHPG-induced Ca^2+^ mobilization (blue box), and was compared to Ca^2+^ mobilization (red box). (C) Bar graphs summarizing the relative contribution of Ca^2+^ mobilization (red), Ca^2+^ influx (blue) and estimated supralinear Ca^2+^ mobilization (black) in both conditions. (D) Ca_Glu_ was not inhibited by U73122. Scale bars indicate 10 sec (horizontal) and 50 nM (vertical).

We estimated Ca_SUP_ by subtracting the sum of Ca_DHPG_ (representing RyR-dependent release; indicated by the red boxes in Figure 7A & 7B) and Ca_Influx_, which was estimated from the glutamate-induced Ca^2+^ transients in the presence of MPEP or ryanodine (indicated by blue boxes, Figure 7A & 7B) from Ca_Glu_ in the control condition (Ca_SUP_ = Ca_Glu_−Ca_DHPG_−Ca_Influx_). In this series of experiments, Ca_DHPG_ was 34.3±10.2% of Ca_Glu_ (n = 6, Figure 7A) and 39.7±12.9 of Ca_Glu_ (n = 4, Figure 7B), which is comparable to the values shown in Figure 4 (39.0±3.6%, n = 74). The estimated Ca_Influx_ was found to be 58.9±10.2% of Ca_Glu_ (n = 6, Figure 7A) in MPEP and 52.4±19.0% of Ca_Glu_ (n = 4, Figure 7B) in ryanodine. Thus, the sum of Ca_DHPG_ and Ca_Influx_ was close to Ca_Glu_ (93.2±10.5% in Figure 7A and 92.1±9.1% in Figure 7B), and Ca_SUP_ was found to be negligible (Figure 7C). Because previous reports have demonstrated the role of IP_3_Rs in synergistic Ca^2+^ release by mGluR and backpropagating APs [17], [18], we tested the effect of U73122 (1 µM) but found no significant effect. Ca_Glu,2_ in the presence of U73122 was 93.6±8.8% of Ca_Glu,1_ (n = 8, Figure 7D), suggesting that the PLC/IP_3_ pathway does not contribute to either Ca_DHPG_ or Ca_SUP_. Taken together, these results suggest that, in the somata of hippocampal neurons, cADPR/RyR-dependent Ca^2+^ mobilization by mGluR5 and Ca^2+^ influx through the L-type Ca^2+^ channels do not interact to generate supralinear Ca^2+^ transients.

## Discussion

We have demonstrated the mechanisms underlying the mGluR5-induced Ca^2+^ mobilization in the somata of hippocampal neurons. Our results indicate that the cADPR signaling pathways are responsible for the mGluR5-induced Ca^2+^ mobilization from ryanodine-sensitive stores. In addition, we found that glutamate-induced Ca^2+^ influx via the L-type Ca^2+^ channels does not interact with mGluR5-induced Ca^2+^ mobilization to cause a supralinear Ca^2+^ increase. These results provide novel insights into the mechanisms for group I mGluR-induced Ca^2+^ mobilization in the somata of hippocampal neurons.

cADPR has long been known to be an endogenous Ca^2+^-releasing messenger [45], [46], [51], and the role of cADPR in neuronal Ca^2+^ signaling has previously been identified [41], [43], [44]. Involvement of cADPR in mGluR-mediated Ca^2+^ signaling was previously demonstrated in midbrain dopamine neurons [19]. This study demonstrated that, in the presence of synaptic blockers (except for mGluR), synaptic stimulation of the dopamine neurons evoked Ca^2+^ waves originating in dendrites 10–50 µm away from the soma, and that the mGluR-induced Ca^2+^ waves were inhibited only when both cADPR and PLC/IP_3_ signaling pathways were inhibited. It was thus concluded that mGluR-mediated Ca^2+^ mobilization involves two pathways mediated by cADPR and IP_3_ in a redundant manner. Our results differ, in that only the cADPR signaling pathway and ryanodine-sensitive stores contributed to Ca^2+^ mobilization by mGluR5 in the somata of hippocampal neurons (Figures 2 & 3). However, these results do not mean that the Ca^2+^ stores in hippocampal neuron somata are insensitive to IP_3_, as muscarinic receptor-mediated Ca^2+^ mobilization was mediated by PLC/IP_3_ signaling pathway (Figure 2). Possibly, Ca^2+^ stores in hippocampal neurons are fundamentally sensitive to both IP_3_ and cADPR, but signaling pathways that regulate these mediators can differ depending on cell types and subcellular localization. It will be of interest to test the contribution of the cADPR-mediated Ca^2+^ releases from RyRs to dendritic Ca^2+^ signaling in the hippocampus.

Dendritic Ca^2+^ signaling induced by group I mGluR has been extensively studied in CA1 hippocampus, and the results obtained in these studies suggest the involvement of the PLC/IP_3_ signaling pathway [17], [18]. Remarkably, large amplitude Ca^2+^ increases induced by repetitive synaptic stimulation, which were considered to be attributable to IP_3_-induced Ca^2+^ release, were precisely confined to the large apical dendrite shaft at the branch point of oblique dendrites [55]. This result indicates that even in dendrites of the same neuron, Ca^2+^ signaling mechanisms are spatially segregated. In the present study, we used acutely dissociated hippocampal neurons with thick apical dendrites of ∼50 µm. We measured Ca^2+^ signals only from somata in response to bath application of mGluR agonist or glutamate. Thus, our results represent somatic Ca^2+^ release mechanisms without interference from dendritic Ca^2+^ signaling mechanisms. We have provided solid evidence that in the somata of hippocampal CA1 pyramidal neurons, cADPR-mediated Ca^2+^ releases from RyRs serve as the predominant mechanism in mGluR-induced Ca^2+^ release. It should also be noted that there has not been a direct examination of somatic Ca^2+^ release machinery despite the fact that somatic Ca^2+^ signals have distinctive roles, such as protein synthesis and gene expression [31], [32]. Further studies are required to test the possibility that dendritic and somatic Ca^2+^ release mechanisms may be distinct from each other in hippocampus.

One of the interesting features reported for mGluR-mediated Ca^2+^ release in dendrites is that Ca^2+^ entry through VGCCs interacts synergistically with IP_3_, and supralinearly increase the GluR-mediated Ca^2+^ release in apical dendrites of hippocampal CA1 neurons [17], [18]. Supralinear Ca^2+^ release by DHPG and either membrane potential depolarization or NMDA receptor activation was also demonstrated in primary cultured hippocampal neurons [13]. However, we showed that cADPR/RyR-dependent Ca^2+^ release by mGluR5 was not supralinearly increased by Ca^2+^ influx in the somata of CA1 pyramidal neurons (Figure 7). This suggests that, unlike IP_3_-dependent Ca^2+^ releases, cADPR-dependent Ca^2+^ release through RyRs is not potentiated by Ca^2+^ influx. However, we still need to consider another type of possible synergism between cADPR/RyR-dependent Ca^2+^ release and L-type Ca^2+^ channels, as demonstrated in previous studies. In NG108-15 cells transfected with mGluRs, direct applications of cADPR enhanced Ca^2+^ influx through L-type Ca^2+^ channels [54]. In addition, dendritic Ca^2+^ transients evoked by back-propagating action potentials, which are mediated by VGCCs, were potentiated by mGluR5-mediated Ca^2+^ release and PKC activation in hippocampal oriens-alveus interneurons [53]. These findings suggested that the RyRs-sensitive Ca^2+^ releases enhance L-type Ca^2+^ channels via PKC-dependent mechanisms. An interesting observation in this study is that the potentiation occurs exclusively in specific microdomains of dendrites; possible absence of similar microdomains in the somata of CA1 pyramidal neurons, which needs to be determined in future studies, would explain the lack of a synergistic interaction found in this study (Figure 7).

In this study, the experiments were performed using acutely dissociated neurons, as dissociated neurons have several advantages in studying signaling mechanisms in the somata. In this preparation, indirect effects or presynaptic components can be excluded. Furthermore, rapid application and wash-out of drugs are guaranteed. It is very difficult to obtain healthy cells by enzymatic dissociation method from rats over 2 weeks old, and therefore we used immature rats (P7-P14). However, glutamate signaling is still developing at this age, and the Ca^2+^ mobilization mechanisms found in the current study may not extend into the somatic mechanism of adult neurons. To exclude this possibility, we examined the DHPG-induced Ca^2+^ release mechanisms in the somata of CA1 pyramidal neurons in brain slices from 4-week-old rats (*data not shown*), and found that the mechanisms were consistent with those found in acutely dissociated immature neurons. Therefore, the mechanisms of mGluR5-induced somatic Ca^2+^ mobilization found in the current study may be extended into at least young adult neurons.

We have demonstrated that AMPA receptors and L-type Ca^2+^ channels, but not NMDA receptors, are responsible for the Ca^2+^ influx by glutamate. It was shown previously that NMDA-dependent Ca^2+^ entry evokes Ca^2+^ increases primarily in spines, which are more concentrated with oblique dendrites [56], [57]. Nakamura et al. [55] also showed that synaptic stimulation evoked Ca^2+^ influx by NMDA receptors exclusively at oblique dendrites, whereas backpropagating APs evoke Ca^2+^ increase at all dendritic locations. Thus, in acutely dissociated neurons that usually lack oblique dendrites the role of NMDA receptors in Ca^2+^ influx should be limited and membrane potential depolarization by AMPA receptors and the opening of L-type Ca^2+^ channels may be responsible for Ca^2+^ influx instead. Another notable finding is that the contribution of Ca^2+^ influx to Ca_Glu_ was larger than that of Ca^2+^ release (Figure 7), signifying the importance of L-type Ca^2+^ channels in somatic Ca^2+^ signaling.

In summary, we investigated the Ca^2+^ mobilization mechanisms by group I mGuRs using acutely dissociated hippocampal neurons. As discussed, the signaling pathways revealed in the current study may represent what occurs in the somata of hippocampal CA1 neurons, and this may be distinct from dendritic Ca^2+^ release machinery, which has been extensively characterized by other groups. The nucleus, as well as other important intracellular organelles, resides in the somata. Therefore, Ca^2+^-dependent molecules regulating cellular excitability and synaptic plasticity may be regulated by the cADPR/RyR-dependent Ca^2+^ release by group I mGluRs in hippocampal CA1 neurons.

## Materials and Methods

### Ethics Statement

Protocols were approved by the Animal Care Committee at Seoul National University (SNU-080107-7). Animal handling was conducted in accordance with national and international guidelines. The number of animals used was minimized, and all necessary precautions were taken to mitigate pain or suffering.

### Preparation of acutely isolated hippocampal neurons

Hippocampal CA1 pyramidal neurons were isolated as described previously [21]. Briefly, 7 to 14-day-old Sprague-Dawley rats (14 to 34 g) were decapitated under pentobarbital anesthesia. The brain was quickly removed and submerged in ice-cold artificial cerebrospinal fluid (ACSF, *see below*) saturated with 95% O_2_ and 5% CO_2_. Transverse hippocampal slices (400 µm thick) were prepared using a vibratome (VT1200, Leica). After a 30 min recovery period at 32°C, the slices were treated with protease type XIV (1 mg/5 ml, Sigma) for 30–60 min, and with protease type X (1 mg/5 ml, Sigma) for 10–15 min at 32°C. The slices were allowed to recover during a 1-hour incubation period at room temperature. The CA1 region was identified and punched out under a binocular microscope (SZ40, Olympus), placed in a recording chamber containing normal Tyrode (NT) solution (*see below*) and mechanically dissociated using a Pasteur pipette to release individual neurons. The dissociated neurons were allowed to adhere to the bottom of the recording chamber for 10–20 min. Cells were identified as pyramidal neurons by their typical large pyramidal-shaped cell body with a thick apical dendritic stump of ∼50 µm under an inverted microscope (IX70, Olympus). The isolation of hippocampal CA1 neurons from PLCβ1 or PLCβ4 knockout mice (generated as described in [35]) was performed as above.

### Solutions and drugs

ACSF contained (in mM): NaCl 125, NaHCO_3_ 25, KCl 3, NaH_2_PO_4_ 1.25, CaCl_2_ 2, MgCl_2_ 1, glucose 10, sucrose 5, vitamin C 0.4, and was bubbled with a mixture of 95% O_2_ and 5% CO_2_ to a final pH of 7.4. NT solution contained (in mM): NaCl 150, KCl 5, CaCl_2_ 2, MgCl_2_ 1, glucose 10, Hepes 10, and was adjusted to pH 7.4 with Tris-OH. To make Ca^2+^-free NT solutions, CaCl_2_ was replaced with equimolar MgCl_2_ and 0.1 mM EGTA. Pipette solutions used for electrophysiology studies contained (in mM) K-gluconate 110, KCl 30, Hepes 20, Mg-ATP 4, Na-vitamin C 4, Na-GTP 0.3, EGTA 0.1 titrated to pH 7.3 with KOH.

(*RS*)-3,5-DHPG, LY367385, MPEP, SKF96365, CNQX, AP-5, ryanodine, TTX were purchased from Tocris. U73122 was purchased from Biomol. Fura 2, Fura 2-AM and 8-NH_2_-cADPR were obtained from Molecular Probes, and ω-conotoxin-GVIA and ω-agatoxin-IVA were from Anygen. All other drugs were purchased from Sigma. Stock solutions of drugs were made by dissolving in deionized water or DMSO according to manufacturer's specifications and were stored at −20°C. On the day of the experiment one aliquot was thawed and used. The final concentration of DMSO in solutions was maintained below 0.1%.

### Calcium measurements

Acutely dissociated hippocampal CA1 neurons were loaded by incubation with 2 µM Fura 2-AM plus 0.01% Pluronic F-127 in NT solution for 10 min at room temperature. For fluorescence excitation, we used a polychromatic light source (xenon-lamp based, Polychrome-IV; TILL-Photonics), which was coupled to the epi-illumination port of an inverted microscope (IX70, Olympus) via a quartz light guide and a UV condenser. Microfluorometry was performed with a 40× water immersion objective (NA 1.15, UAPO 40× W/340, Olympus) and a photodiode (TILL-Photonics).

### Calibration of Ca^2+^ measurements

Calibration parameters were determined using in vivo calibration as described in [58]. The effective dissociation constant of Fura 2 (*K*_eff_) was calculated from *K*_eff_ = [Ca^2+^](*R*_max_−*R*_int_)/(*R*_int_−*R*_min_), where [Ca^2+^] was entered as 231 nM (assuming a dissociation constant (*K*_d_) of BAPTA of 222 nM at pH 7.2). The estimated *R*_min_, *R*_max_ and *K*_eff_ (µM) measured using an inverted microscope were typically 0.27, 3.95 and 0.93, respectively. A standard two-wavelength protocol was used for fluorescence measurement of cells. Fluorescence intensity was measured at 1 Hz with double wavelength excitation at 340 nm (*F*_340_) and 380 nm (*F*_380_). The ratio *R* = *F*_340_/*F*_380_ was converted to [Ca^2+^] values using the equation [Ca^2+^] = *K*_eff_ (*R*−*R*_min_)/(*R*_max_−*R*).

### Electrophysiology

Current clamp recordings of membrane potential were performed using an EPC-10 amplifier (HEKA Elektronik) at room temperature. Membrane potentials were recorded from acutely dissociated hippocampal CA1 neurons in a conventional whole cell configuration at a sampling rate of 10 kHz filtered at 1 kHz. Data were acquired using an IBM-compatible computer running Pulse software v8.67 (HEKA Elektronik). The patch pipettes were pulled from borosilicate capillaries (Hilgenberg-GmbH) using a Narishige puller (PC-10, Narishige). The patch pipettes had a resistance of 3–5 megaohms when filled with above-mentioned K-based pipette solutions.

### Single cell electroporation

The loading of heparin and 8-NH_2_-cADPR was performed by single cell electroporation. Micropipettes were pulled as described above and were filled at their tips with NT solutions containing heparin (20 mg/ml) or 8-NH_2_-cADPR (100 µM) plus Alexa Fluor-488 (200 µM, Molecular Probes). Micropipettes were controlled by a micromanipulator (Burleigh) to reach cells, and square electric pulses generated with an electroporator (Axoporator 800A; Molecular Devices/MDS Analytical Technologies) were applied to transfer the mixture into the cells.

### Data analysis

Data were analyzed using IgorPro (version 4.1, WaveMetrics) and Origin (version 6.0, Microcal) software. Statistical data are expressed as the mean ± S.E., where *n* represents the number of cells studied. The significance of differences between the peaks was evaluated using a Student's *t*-test with confidence levels of *p*<0.01 (**) and p<0.05 (*).
